# Differences in the cargos and functions of exosomes derived from six cardiac cell types: a systematic review

**DOI:** 10.1186/s13287-019-1297-7

**Published:** 2019-06-27

**Authors:** Ming-yue Xu, Zhi-shuai Ye, Xian-tao Song, Rong-chong Huang

**Affiliations:** 1 Department of Cardiology, The First Affiliated Hospital of Dalian Medical University, 222 Zhongshan Road, Dalian, 116011 People’s Republic of China; 2 Department of Cardiology, Beijing Friendship Hospital, Capital Medical University, 95th Yong An Road, Xuan Wu District, Beijing, 100050 People’s Republic of China; 3 Department of Cardiology, Beijing Anzhen Hospital, Beijing Institute of Heart, Lung and Blood Vessel Disease, Capital Medical University, 2 Anzhen Road, Beijing, 100029 People’s Republic of China

**Keywords:** Exosomes, MicroRNA, Cardiovascular disease (CVD), Cardiac cells

## Abstract

Exosomes are bilayer membrane vesicles with cargos that contain a variety of surface proteins, markers, lipids, nucleic acids, and noncoding RNAs. Exosomes from different cardiac cells participate in the processes of cell migration, proliferation, apoptosis, hypertrophy, and regeneration, as well as angiogenesis and enhanced cardiac function, which accelerate cardiac repair. In this article, we mainly focused on the exosomes from six main types of cardiac cells, i.e., fibroblasts, cardiomyocytes, endothelial cells, cardiac progenitor cells, adipocytes, and cardiac telocytes. This may be the first article to describe the commonalities and differences in regard to the function and underlying mechanisms of exosomes among six cardiac cell types in cardiovascular disease.

## Introduction

Cardiovascular disease (CVD) is a major cause of morbidity and mortality worldwide [1]. Although percutaneous coronary intervention and coronary artery bypass grafting are advanced and precise strategies to regenerate heart tissues, these techniques are insufficient to rescue injured myocardiocytes and delay the progression of heart failure. Many studies have reported that human cardiomyocytes (CMs) can be regenerated; however, the regenerative capacity of these cells is very limited. Thus, alternative technologies are needed to promote cardio-regeneration and delay the progression of CVD.

Stem cell therapy is considered an ideal solution for the replacement of damaged cells, as exosomes derived from stem cells and various paracrine mechanisms can reduce the risk of rejection and improve survival of transplanted stem cells [2].

Extracellular vesicles, which include exosomes (diameter, 30–100 nm), microvesicles (100–1000 nm), and apoptotic bodies (500–4000 nm), differ in terms of biological sources, secretion methods, markers, and contents (Table 1). Exosomes, which are bilayer membrane vesicles that were first extracted from sheep serum in 1979, are formed by intracellular lysosomal microparticles and can be extracted from mammalian blood, urine, and ascites for diagnostic and prognostic evaluations [2, 3]. Exosomes contain a variety of surface protein receptor analogues, biomarkers, such as P-selectin and integrins, lipids, nucleic acids, noncoding RNAs, especially microRNAs (miRNAs), and transcription factors. These molecules are cell-type specific, and expression levels are usually dependent on the physiological and pathological conditions of the cell [3]. Exosomes mediate cell-to-cell communication, stimulate or inhibit the activities of target cells, and impact various biological processes. Exosomes from different types of cardiac cells participate in the processes of angiogenesis and cell migration, proliferation, apoptosis, hypertrophy, and regeneration. In a state of hypoxia, the content [4–6] and the quantity [7, 8] of exosomes in the circulation significantly change, suggesting the release of specific exosomes into body fluids by the injured myocardium.

**Table 1:**
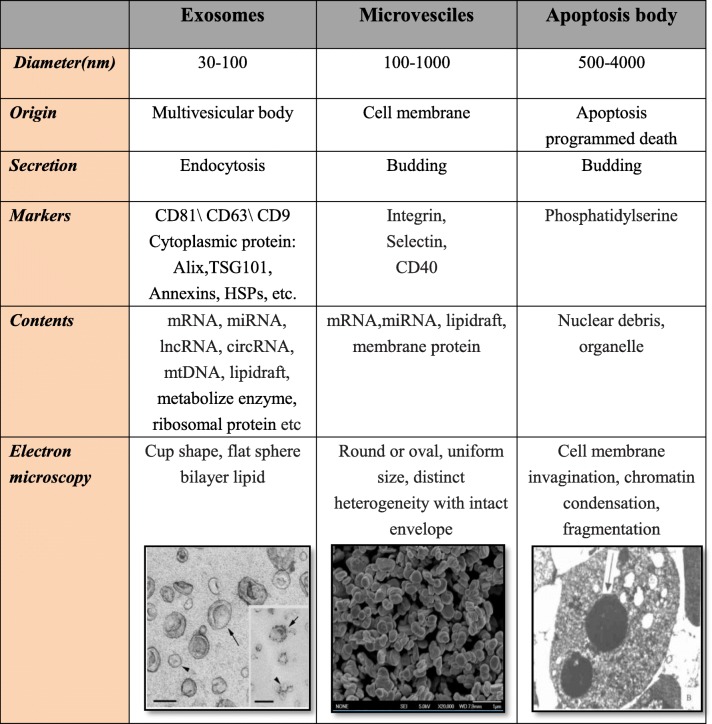
Difference of three extracellular vesicles

**Fig. 1:**
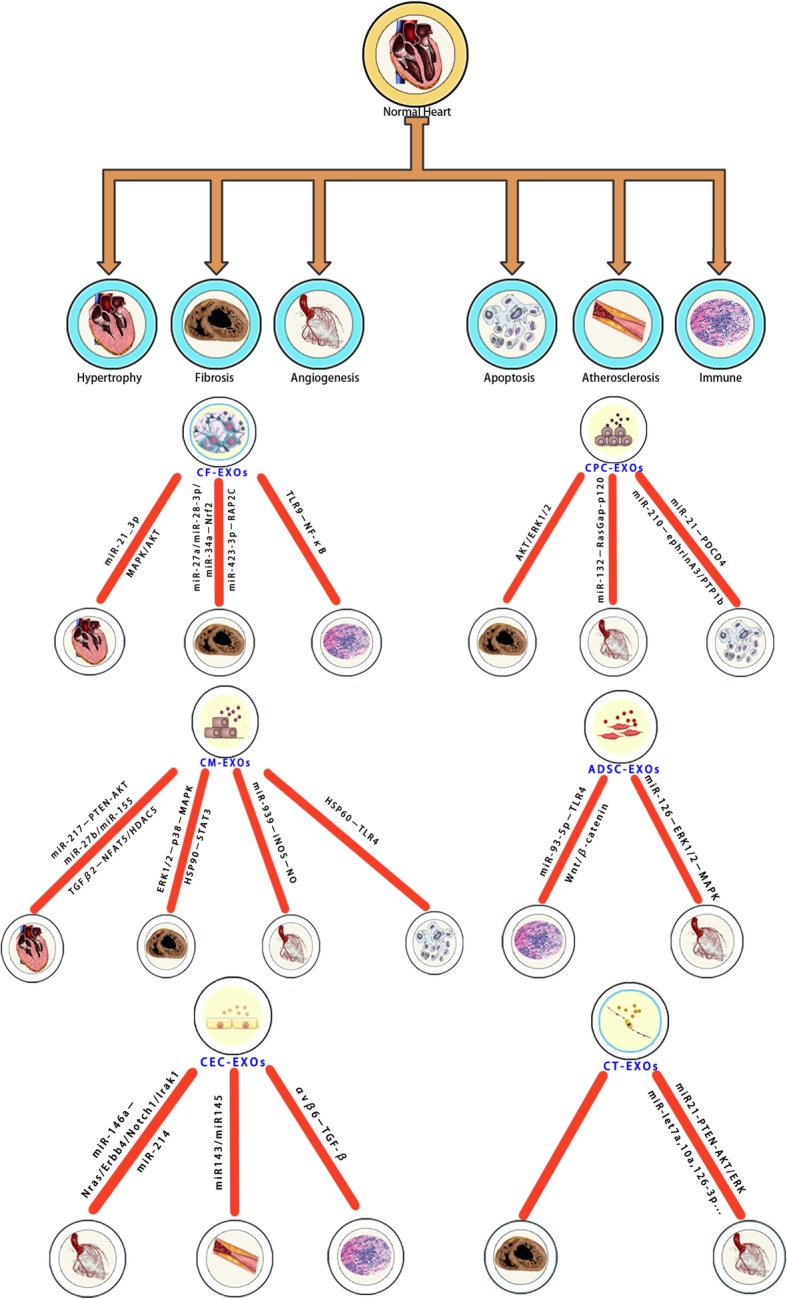
Functions and mechanism of exosomes derived from six cardiac cell types

In CVD, the exosomes of cardiac cells facilitate the exchange of molecular signals and vectors to activate target molecules for the regulation of inflammatory factors, which can eventually promote cardiac regeneration and function [3, 9]. In this article, the roles of the exosomes of six types of cardiac cells were investigated and compared (Fig. 1). A shared key aspect of these cells was the mediation of information exchange by the release of the cargo of exosomes. However, differences in the mechanisms underlying exosome release by these six cell types remain unclear.

### Cardiac fibroblast (CF)-derived exosomes (CF-EXOs)

CFs account for about 60–70% of all normal cardiac cells and about one third of the volume of the normal heart. CFs produce an extracellular matrix that supports the CMs and participates in post-injury repair by regulating the proliferation and migration of cardiac cells. Ischemia, pressure, and volume overload induce hypertrophic cellular responses mediated by crosstalk among fibroblasts, CMs, endothelial cells (ECs), and inflammatory cells via extracellular vesicles [10]. Proteomic analysis of CFs from neonatal rats revealed that under hypoxic conditions, CF-EXOs selectively upregulated signaling activities and the expression levels of proteins associated with the extracellular matrix, as compared with normoxic conditions [11], suggesting that CF-EXOs are involved in many functional pathways.

#### Cardiac hypertrophy

Bang et al. [12] conducted the first substantial study of the roles of miRNAs in exosomes in the crosstalk between CFs and CMs. MiR-21_3p of CF-EXOs was found to migrate to CMs via internalization by endocytosis or a receptor-mediated mechanism, which eventually leads to cardiac hypertrophy. As a possible explanation, miR-21_3p regulates the expression of SORBS2 and PDLIM5, which are functional and structural proteins of CMs. Stimulation of CFs with angiotensin II increased the secretion of miR-21_3p by CF-EXOs. Similarly, treatment of CFs with miR-21_3p inhibitors downregulated angiotensin II levels and reduced the area of cardiac hypertrophy via upregulated activation of the renin-angiotensin system [12, 13].

#### Antifibrotic effects of CF-EXOs

In a mouse model of chronic heart failure (CHF), exosomes from CFs and CMs were found to contain relatively higher levels of miR-27a, miR-28-3p, and miR-34a, which repress the translation of Nrf2 [14]. The Nrf2/ACE signaling pathway plays an antioxidant role in myocardial cell injury and delays the processes of ventricular remodeling and cardiac dysfunction [15]. In a seminal study in this area of clinical research, Wei et al. [16] separated exosomes from the plasma of CHF patients and controls and found that the exosome cargo included mitochondrial DNA, which triggered an inflammatory response via the TLR9-IRAK1-TRAF6-I_κ_B_α_-NF-κB pathway. These results provide new perspectives for the application of CF-EXOs for the intervention and treatment of CHF during the remodeling and inflammatory processes.

#### Cardioprotective effects of CF-EXOs

Some studies have shed light on the functions of CF-EXOs in ischemia-reperfusion injury. For example, Abrial et al. [17] reported that in a rat model of myocardial infarction (MI), the administration of CF-EXOs resulted in a 25% reduction in infraction size, as compared to controls (58 ± 2% vs. 30 ± 2%, respectively), and the presence of CFs in co-culture increased the viability of CMs following hypoxia-reoxygenation injury in a paracrine-dependent manner. In a more in-depth study, Hui et al. [18] found that CF-EXOs had a protective function in CMs both in the acute phase of MI and ischemia post-conditioning through the miR-423-3p/RAP2C pathway.

Although there have been many reports of mesenchymal stem cell (MSC) transplantation for the treatment of ischemic heart disease (IHD), relatively few studies have investigated the roles of CF-EXOs. These studies by Abrial et al. [17] and Hui et al. [18] have undoubtedly provided important reference values for CF-EXOs for the treatment of IHD. Nonetheless, further large-scale animal and human studies are needed to confirm the safety and validity of CF-EXO-based treatments for IHD.

### Cardiomyocyte-derived exosomes (CM-EXOs)

A variety of studies have established that CM-EXOs contain an abundance of miRNAs that can be used to assess the degree of myocardial injury or recovery and can potentially serve as new diagnostic and prognostic biomarkers.

#### Cardiac hypertrophy

Gennebäck et al. [19] proposed an explanatory mechanism of CM-EXO-dependent modulation of the proliferation, development, and hypertrophy of cardiac cells. In this study, HL-1 cells derived from the hearts of adult mice were pretreated with TGF-b2 and PDGF-BB. Then, the exosomes were harvested from each group of cells by centrifugation. Of the differentially expressed transcripts, NFAT5 [20] and HDAC5 [21] are involved in cellular proliferation and hypertrophic signaling. Moreover, the miRNAs as cargo of CM-EXOs were found to play important roles in cardiac hypertrophy. For example, miR-217 expression was upregulated in the plasma of CHF patients and cardiac tissue samples, demonstrating that miR-217 promoted cardiac hypertrophy via the PTEN-AKT pathway [22]. Another study of a mouse model of heart failure found that miR-27b participated in cardiac hypertrophy via the TGF-β1-PPARγ pathway and miR-155 had the same effect via Jaird2 signaling [23, 24]. Hence, targeting of these miRNAs as signal biomarkers may have clinical potential to suppress cardiac hypertrophy and CHF.

#### Cardiac repair

CMs released most differential exosomes in response to ischemic stress. Clinically, exosomes derived from the plasma of MI patients had greater activities in the proliferation and migration of ECs and tube formation via the miR-939-iNOS-NO pathway [25]. In an animal study, Gupta and Knowlton [26] demonstrated that in Sprague-Dawley rats, the cargo of CM-EXOs included HSP60, HSP90, and HSP70. Within 2 h of hypoxia, the amount of HSP60 released from CM-EXOs had increased, while HSP60 was released into the extracellular space, where it combined with TLR-4 as a danger signal of the immune system, which leads to myocardial cytotoxicity and apoptosis of cardiac cells [27]. Under hypoxic conditions, most HSP60 was tightly attached to the membranes of the CM-EXOs, which then acted as a barrier to isolate CMs from the high level of HSP60 in the extracellular space, resulting in the alleviation of apoptosis and reduced toxicity of cardiac cells [28].

#### Antifibrotic effects

Notably, unlike CM-EXO-HSP60, which is involved in cardiac ischemia, CM-EXO-HSP90 regulates collagen synthesis and fibrosis in renal artery-ligated rats. By stimulating IL-6, CM-EXO-HSP90 takes part in both the p53 and STAT-3 pathways in fibroblasts [29]. This study is the first to propose a possible role of CM-EXO-HSP90 in the regulation of IL-6 synthesis and biphasic IL-6-induced activation of STAT-3 in vitro. However, in vivo trials are needed to identify the functions of these cytokines. Besides, CM-EXO-HSP20 was found to alleviate fibrosis and promote angiogenesis via the Tsg101 pathway in diabetic mice [30]. Other antifibrotic effects of CM-EXOs were tested in patients with dystrophin-deficiency disease (i.e., Duchenne muscular dystrophy). In these patients, the cargo of exosomes from induced pluripotent stem cells that were directionally differentiated to CMs had activities against apoptosis and conveyed cardioprotective effects in the form of delayed cardiac fibrosis and cardiomyopathy [31]. These relationships can be partly be explained by CM-EXO-mediated ERK1/2-p38-MAPK signaling. These results present a therapeutic target of interest for the acute protection of the dystrophin-deficient heart against stress-induced injury.

CMs can release cytokines, growth factors, and exosomes. The cargo of CM-EXOs contains various miRNA molecules, including miR-1, miR-133a/b, miR-208a, and miR-499 [6, 32], which are specifically expressed in CMs. The expression levels of these miRNAs in the blood are significantly increased upon impairment of CMs and can be detected earlier than troponins. CM-EXOs contain a basic package of transcripts and growth factors that can be used to intervene in transcription and signaling pathways involved in cell hypertrophy and fibrosis formation, as cardioprotective effects.

### Cardiac endothelial cell (CEC)-derived exosomes (CEC-EXOs)

#### The roles of CEC-EXOs in angiogenesis

CEC-EXO-miR-214 regulates cell migration and angiogenesis by silencing mutations in adjacent target cells and causing dysregulation of capillary dilatation. Consistently, CEC-EXOs from miR-214-knockout ECs failed to conduct these functions [33]. Halkein et al. [34] confirmed the anti-angiogenesis effect of CEC-EXOs in a model of peripartum cardiomyopathy (PPCM) by injecting 16-kDa prolactin and found that miR-146a was mostly expressed in ECs and attenuated angiogenesis by downregulation of Erbb4, Nras, Notch1, and Irak1 signaling. Although two authoritative articles reported that miRNAs embody two opposite roles in promoting and inhibiting angiogenesis, both delay the clinical progression of PPCM. MiRNAs in CEC-EXOs promote angiogenesis and cell migration and inhibit angiogenesis by delaying the deterioration of cardiac function in PPCM. From this point of view, EC-EXOs are extremely important in the regulation of various cellular functions.

#### Cardioprotective effects

Following the Framingham Heart Study, Amabile et al. [35] studied endothelial exosomes in 844 individuals without CVD in the Framingham Offspring cohort study. The results of flow cytometry revealed that surface biomarkers on endothelial exosomes were strongly correlated with cardiometabolic risk factors. For example, CD144^+^ expression was significantly upregulated in patients with hypertension and the elevated expression levels of both CD144^+^ and CD31^+^/CD41 were correlated with a greater body mass index, larger waist circumference, lower high-density lipoprotein cholesterol levels, and higher triglyceride levels. This study provides a new assessment tool for the prevention and prediction of CVD, although other mechanisms underlying metabolic and lipid abnormalities that lead to the differentiation of biomarkers on endothelial-derived exosomes must be further explored.

Other than markers of risk factors associated with CVD, CEC-EXOs also play roles in the prevention of atherosclerosis. Drawing on an extensive range of sources, many studies have investigated the mechanisms of miRNAs underlying the regulation of transcription factors. For example, Krüppel-like factor 2 (KLF2)-miR-143/145 [36] was identified as the main atheroprotective pathway of CEC-EXOs. By injecting KLF2-transduced and KLF2-transducted ECs with locked nucleic acid-modified oligonucleotides to silence miR-143/145 to high-fat diet ApoE^−/−^ mice, exosomes derived from KLF2-expressing ECs were found to protect against atherosclerotic lesions in the aorta.

#### Anti-inflammatory effects

In a study by Song et al. [37], CEC-EXOs exposed to lipopolysaccharides tended to express integrin αvβ6, which resulted in the proliferation of T cells via the αvβ6-TGF-β pathway, while other studies have confirmed the involvement of TGF-β in the formation and growth of atherosclerotic plaques. A clinical study found that serum HSP70 levels are negative correlative to CVD, especially hypertension, atherosclerosis, and coronary vascular disease [38], and Zhan et al. [39] revealed a mechanism in which oxidative low-density lipoprotein and homocysteine induce HSP70 release from EC-EXOs, which activates monocytes and promotes adhesion of monocytes to ECs. HSP70 on EC-EXOs provides a new direction to further understand the mechanisms of vascular endothelial regulation. Research on exosomes derived from human umbilical vein ECs or human coronary artery ECs showed that these EC-EXOs can modulate inflammation and regulate monocyte activation and migration. In vitro, EC-EXOs were found to suppress monocyte activation by enhancing immune-modulatory responses and diminishing pro-inflammatory responses, consistent with an in vivo study by injecting EC-EXOs to rats [40]. EC-EXOs translated miR-10a from ECs to monocyte to inhibit inflammatory responses via NF-κB signaling and inhibiting the activation of pro-inflammatory genes, such as BTRC, MAP3K7, and IRAK4 [40, 41]. Other researchers have found that CE-EXOs not only regulate inflammation at the miRNA level, but also play a role at the protein level.

Notably, past studies of CEC-EXOs have mainly focused on angiogenesis and vascular injury. However, CEC-EXOs, CMs-EXOs, and CF-EXOs have various functions in CVD.

### Cardiac progenitor cell (CPC)-derived exosomes (CPC-EXOs)

CPCs, first discovered in the rat heart, have the ability to differentiate into major cardiac cells in the myocardium and cardiac vessels. Progenitor cells, also known as precursor cells, reside between stem cells and adult cells. CPCs can only exist for several days in experimental mice, but for several weeks in the human fetus, suggesting an important role in the formation of the human heart. Studies have found that CPC-EXOs can improve cardiac function after injury. The specific functions and mechanisms of CPCs are described in the following sections.

#### Cardiac repair and regeneration

Relatively few studies have compared the efficacies of different cell-derived exosomes in CVD. Of these, a Swiss research team found that CPC-EXOs were more therapeutically efficient than MSC-EXOs in a mouse model of MI [42]. Both types of exosomes enriched miR-146a-3p, miR-132, and miR-181a and stimulated blood vessel formation. However, the main differences have been identified in in vivo trials. For example, in a permanent coronary ligation model, CPC-EXOs were found to enhance blood vessel density within the infarct area and in an ischemia/reperfusion (I/R) model, as injection of CPC-EXOs increased the left ventricular ejection fraction. Another study found that PPAP-A regulated the function of CPC-EXOs via the PPAP-A-IGF1-Akt/ERK1/2 pathway, which may explain the in vivo benefit. The results of microarray analysis found that under hypoxic conditions, the cargo of CPC-EXOs contained 11 differential miRNAs, which enhanced tube formation of ECs and decreased TGF-β stimulation of fibroblasts [43]. To elucidate the functions of CPC-EXOs in an animal model of MI, Gray et al. [43] injected rats with CPC-EXOs under hypoxic conditions. As predicted, under hypoxic conditions, CPC-EXOs improved cardiac function and delayed fibrosis. Although these differentially expressed miRNAs in CPC-EXOs were thought to be responsible for these effects, the authors failed to identify downstream signaling factors. miRNAs, such as miR-17, miR-210, miR-292, and miR-133a, as paracrine factors of CPC-EXOs, were all correlated with cardioprotective effects and the alleviation of fibrosis, although only miR-133 was verified in the MI model [44].

#### Anti-apoptosis effects

In an in vitro study, Xiao et al. [45] demonstrated that upon exposure to oxidative stress, apoptosis of H9C2 cardiac cells was accelerated and the production of CPC-EXOs was enhanced, while the addition of miR-21 to the cargo of CPC-EXOs reversed these trends and protected CMs from apoptosis via the PDCD4 pathway. In an in vivo study, Barile et al. [46] obtained CPCs and CFs from the atrial appendage explants of patients who underwent heart valve surgery. By injecting human CPC-EXOs and CF-EXOs to an MI model, as compared with CF-EXOs, the CPC-EXO-treated group exhibited less CM apoptosis, enhanced angiogenesis, and improved left ventricular ejection fraction (0.8 ± 6.8% vs. − 21.3 ± 4.5%; *p* < 0.05). In an in vitro study of CPC-EXOs, miR-210 targeted the ephrin A3 gene and PTP1b inhibited apoptosis of CMs, while miR-132 and RasGAP-p120 enhanced tube formation of CECs. However, as compared to CPC-EXOs, there is less miRNA cargo in CF-EXOs, which can explain why CPC-EXOs are more effective against apoptosis and angiogenesis. Another study confirmed that CPC-EXOs had more beneficial effects than CPCs when transplanted in an MI mode [47], as the CPC-EXOs group had less scarring and greater CM proliferation.

Taken together, the results of these studies confirmed that human and mouse CPC-EXOs can reduce scar size and promote cardiac repair by alleviating apoptosis and enhancing angiogenesis following MI. As the most exciting result, transplantation of CPC-EXOs had a more curative effect than CF-EXOs, MSC-EXOs, and CPCs on cardiac repair in vivo. A study of circulation found, for the first time, that donor age and oxygen content in the microenvironment significantly altered the efficacy of human CPC-EXOs [48]. Also, specific miRNA expression in CPC-EXOs enabled more efficient function than other cell-derived exosomes, drugs, and inhibitors targeting miRNAs, suggesting that targeting of signaling molecules will pave the way for new therapies to protect cardiac cells following MI.

### Adipocyte-derived exosomes

Under physiological conditions, cardiac tissues contain relatively few adipocytes. As an important endocrine organ, adipose tissue secrets a variety of peptide hormones and cytokines via paracrine- and autocrine-mediated mechanisms. As another important function, adipocytes also secrete exosomes [49]. The heart contains a small amount of adipose tissue located in the epicardium, while under pathological conditions, there is a large amount of adipose tissue. Most studies in this area have mainly focused on the secretion of exosomes by visceral adipocytes or adipose-derived stem cells (ADSCs). ADSCs are stem cells with multi-differentiation potential that are capable of self-replication and cloning. The cargo of ADSC-derived exosomes (ADSC-EXOs) contains proteins, immunological factors, mRNA, and miRNAs, which are involved in metabolism, as well as the activities of immune cells [50].

#### Angiogenesis

Kang et al. [51] pointed out that miR-31 was notably elevated in the cargo of ADSC-EXOs following preconditioning with a medium that promotes endothelial differentiation. HIF-1 was identified as the target of miR-31, which may explain the anti-angiogenic function of ADSC-EXOs [51]. This research paves the way for the use of engineered microvesicles as an alternative approach for the treatment of ischemic diseases. In a clinical study, extracellular vesicles collected from the adipose tissues of obese patients had impaired angiogenic potential. Besides VEGF and MMP-2, the miR-126-Spred1-ERK1/2-MAPK signaling pathway was found to play a major role in angiogenesis [52].

#### Cardiac repair

According to Luo et al. [53], gene editing of exosomes may be realized in the near future. The results of experiments using a rat model of acute MI (AMI) suggested that miR-126 enhanced ADSC-EXOs as cardiac protector cytokines in CMs. This source of exosomes alleviated fibrosis and apoptosis, and by detecting the expression levels of the inflammatory factors IL-6, IL-1β, and TNF-α, the researchers also found that exosomes attenuated the inflammatory responses in a rat model of AMI. Two other studies reported that ADSC-EXOs attenuated AMI-induced myocardial injury via the miR-93-5p/TLR4 and Wnt/β-catenin signaling pathways [54, 55]. Cui et al. identified that Wnt/β-catenin signaling pathways also play a role in adipose-derived MSCs exosomes (ADMSC-EXOs) in rats models of myocardial I/R and hypoxia/reoxygenation (H/R) injury model [55]. In this article, ADMSC-EXOs significantly reduced the serum level of CK-MB as well as the apoptosis of CMs by downregulating some apoptotic factors.

Liu et al. found that ADSC-EXOs significantly attenuated I/R-induced production of reactive oxygen species by reducing the expression levels of proteins associated with apoptosis (P-p53, PUMA) and hypertrophy (Ets-1, atrial natriuretic peptide) in a mouse model [56]. In addition to angiogenesis, cardiac repair and the anti-inflammatory functions mentioned above, ADSC-EXOs reported to play roles in decreased the expression of proteins associated with apoptosis and hypertrophy of CMs in a mouse model of cardiac I/R.

Other studies have found that pretreated, as compared with untreated, ADSC-EXOs under hypoxic conditions have better effects on cell migration and capillary network formation, indicating that ADSC-EXOs can secrete extra factors with protective and repair functions in response to injury [57]. In summary, both ADSC-EXOs and ADMSC-EXOs can repair the myocardium in terms of ischemia, hypoxia, and ventricular remodeling. In the field of regenerative medicine, other than cell and non-cell treatment methods, artificial editing or intervention of ADSC-EXOs in vitro is expected to achieve better therapeutic effects, which will undoubtedly provide new ideas for future research in CVD and regenerative medicine.

### Cardiac telocyte (CT)-derived exosomes (CT-EXOs)

CTs, formerly called interstitial Cajal-like cells, are interstitial cells in the human epicardium and myocardium that were first identified by transmission electron microscopy in 2010 [58]. CTs were found to be widely distributed in almost all organs and tissues, including the heart [59]. Notably, CT numbers were reduced in the myocardium of CVD patients, which may be involved in the abnormal three-dimensional structure of cells and interfere with signal transduction between cardiac cells [60]. CTs have extremely thin and long telopodes, alone with complex protruding three-dimensional structures that may support and protect the biological function of certain cells in the heart. In particular, the cargo of CT-EXOs contains cell-specific proteins, lipids, and nucleic acids, which participate in intercellular signal transduction and the functional regulation of multiple adjacent cells [61].

#### Angiogenesis

The functions of CT-EXOs include the promotion of ECs proliferation and migration, and the formation of capillary-like structures in a co-culture system, as these effects were ameliorated upon exosome depletion [62]. Zhao et al. injected CTs from rats and phosphate-buffered saline (PBS) to MI model rats and found that transplantation of CTs was able to significantly decrease the infarct size and improved cardiac function for 14 weeks after MI [63]. Moreover, the authors revealed that this beneficial effect was mainly attributed to increased cardiac angiogenesis and the paracrine function of CTs. Another study provided some direct evidence of CT-EXOs taking part in angiogenesis at 30 days after MI in rats [64]. Electron microscopy showed the CTs were connected with ECs by both directly and indirectly. The expression levels of VEGF and NO, as determined by immunocytochemical analysis, in MI, as compared with the normal heart, indicate that CTs may promote or modulate angiogenesis via VEGF and/or NO secretion. In addition, the cargo of CT-EXOs include a series of pro-angiogenic miRNAs (e.g., let-7e, 10a, 21, 27b, 100, 126-3p, 130a, 143, 155, and 503) that regulate the activities of neighboring ECs by the promotion of angiogenesis and infarct myocardial repair via a paracrine-mediated mechanism [64]. Also, miRNA-21 mediated angiogenesis via the PTEN-AKT/ERK1/2-HIF1α/VEGF pathway [65], which may be similar to CT-EXOs during neo-angiogenesis after MI.

#### Cardiac regeneration and repair

An increase in the number of CTs enhanced the secretion of exosomes, as observed around the CTs in the post-infarcted border of the heart [64]. In an in vivo study, CT-EXOs and PBS, as a control, were injected into two groups of MI rats. Echocardiography showed less cardiac fibrosis and reduced collagen deposition in the CT-EXO-treated group, demonstrating that CT-EXOs improved cardiac function and promoted cardiac repair [62]. This result suggests that CT-EXOs may be considered as a new treatment option for myocardial repair and improved cardiac function after MI. Also, CTs have been found within the stem cell niche of several organs and these neighboring stem cells were regulated by miRNAs loaded on CT-EXOs both in vivo and in vitro [66].

Stem cell transplantation has been proven to be effective. Since CTs are increased after injury, more CT-EXOs are secreted in the marginal zone of infarcted myocardium. However, it remains unclear whether CT-EXOs accelerate myocardial repair and promote angiogenesis during stem cell transplantation for MI. The roles of CT-EXOs have been confirmed in angiogenesis and cardiac repair after MI, although further studies are needed to elucidate the mechanisms and pathways responsible for the communication between CT-EXOs and neighboring cells.

### Vascular smooth muscle cell (VSMC)-derived exosomes (VSMC-EXOs)

Though mainly located in vascular tissue, rather than cardiac tissues, VSMCs play vital roles in CVD. Research on VSMC-EXOs has mainly focused on vascular calcification during atherosclerotic chronic kidney disease and diabetes, which both increase the risk of CVD. Activation of VSMCs and macrophage invasion promote atherosclerotic plate formation [67]. The cargo proteins and miRNAs of VSMC-EXOs take part in CVD. For example, MiR-155 in VSMC-EXOs is transferred to ECs to destroy tight junctions and the integrity of endothelial barriers, leading to an increase in endothelial permeability and enhanced atherosclerotic progression [68]. Also, miR-211/222 from human aortic smooth muscle cell-derived exosomes (HAoSMC-EXOs) also play roles in cardiovascular dysfunction and atherosclerosis by modulating autophagy of ECs via the PTEN/Akt signaling pathway [69]. The cargo of exosomes is released in clarified VSMCs as a result of the loss the contractile VSMC phenotype and mineral imbalance, which promotes calcification [70, 71]. An article on circulation research has shed light on pulmonary artery smooth muscle cell-derived exosomes (PASMC-EXOs) and found that miR-143-3p in PASMC-EXOs elevated and altered cell migration and apoptosis in both calf models of pulmonary arterial hypertension(PAH) as well as in samples from PAH patients [72]. PASMC-EXOs and their cargo may act as a crucial paracrine signaling mediator during the remodeling of the pulmonary vasculature.

## Conclusion

The aim of this article is to describe the functions and possible signaling pathways of CF-EXOs, CM-EXOs, CEC-EXOs, CPC-EXOs, ADSC-EXOs, CT-EXOs, and VSMC-EXOs (Table 2). Although nearly all exosomes positively regulate apoptosis and angiogenesis in cardiac tissues, only CF-EXOs, CEC-EXOs, and ASDC-EXOs have roles in the immune and inflammatory processes in MI via TLRs, and only CF-EXOs, CM-EXOs, and ADSC-EXOs inhibit cardiac fibrosis in MI and CHF. Moreover, CM-EXOs were confirmed to attenuate cardiac hypertrophy in CHF via angiotensin II and HSP-related mechanisms. CEC-EXOs and VSMC-EXOs have atheroprotective functions and thus are considered as cardiometabolic risk factors that may be beneficial to CVD. Normal CEC-EXOs promote angiogenesis, while those from PPCM patients inhibit angiogenesis, suggesting that CEC-EXOs have the strong adaptive capacity and self-regulating function in the regulation of cardiac repair and delayed cardiomyopathy. The curative effect of CPC-EXOs is better than that of CPC, MSC-EXOs, and CF-EXOS, which can be explained by the specific miRNA and cytokines in the cargo of CPC-EXOs. Regulation of miRNAs in ADSC-EXOs and pretreated ADSC-EXOs under hypoxic conditions both enhanced the curative effect against AMI, suggesting the usefulness of engineered exosomes as an alternative treatment for ischemic diseases and regeneration medicine. Different sources of smooth muscle cells and their exosomes (i.e., VSMC-EXOs, HAoSMC-EXOs, and PAMSC-EXOs) contain various miRNAs that participate in the pathology of diseases. Thus, engineered miRNAs in exosomes present new therapeutic targets.

**Table 2:**
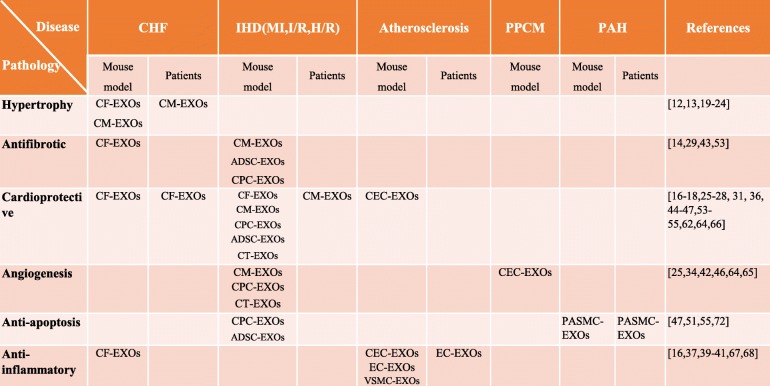
The involvement of different exosomes in cardiac pathological process

Exosome transplantation is an efficient treatment option for CVD, as several basic and clinical studies have confirmed decreased immune rejection and improved survival, as compared with cell transplantation. miRNAs and proteins released from various cardiac cell exosomes regulate target gene expression and cell function, which also play important roles in alleviating cardiac hypertrophy, dysfunction, and fibrosis, while promoting angiogenesis, post-infarction myocardial repair, and anti-atherosclerosis progression. In conclusion, cardiac cell exosomes and miRNAs may be used as biomarkers for the diagnosis and prognosis of CVD. The specific targeting of miRNAs to molecules involved in various signaling pathways offers new therapeutic options for CHF, ischemia, and cardiomyopathy. The gold standard for assessment of CVD must be efficient and beneficial to patients with low complication rates, thus further clinical trials are needed to confirm the effects of exosomes for use in targeted interventional medicine.

## Data Availability

Not applicable

## Abbreviations

ADMSC-EXOs: Adipose-derived mesenchymal stem cell exosomes
ADSC-EXOs: Adipose-derived stem cells exosomes
ADSCs: Adipose-derived stem cells
AMI: Acute myocardial infarction
CEC: Cardiac endothelial cells
CEC-EXOs: Cardiac endothelial cell-derived exosomes
CF: Cardiac fibroblast
CF-EXOs: Cardiac fibroblast-derived exosomes
CHF: Chronic heart failure
CM-EXOs: Cardiomyocyte-derived exosomes
CMs: Cardiomyocytes
CPC: Cardiac progenitor cell
CPC-EXOs: Cardiac progenitor cell-derived exosomes
CT: Cardiac telocyte
CT-EXOs: Cardiac telocyte-derived exosomes
CVD: Cardiovascular disease
EC-EXOs: Endothelial cell-derived exosomes
ECs: Endothelial cells
H/R: Hypoxia/reoxygenation
HAoSMC-EXOs: Human aortic smooth muscle cell-derived exosomes
I/R: Ischemia/reperfusion
IHD: Ischemic heart disease
MI: Myocardial infarction
MSC: Mesenchymal stem cell
PAH: Pulmonary arterial hypertension
PASMC-EXOs: Pulmonary artery smooth muscle cell-derived exosomes
PBS: Phosphate-buffered saline
PPCM: Peripartum cardiomyopathy
VSMC: Vascular smooth muscle cell
VSMC-EXOs: Vascular smooth muscle cell-derived exosomes
